# Relationship between Human Papillomavirus Status and the Cervicovaginal Microbiome in Cervical Cancer

**DOI:** 10.3390/microorganisms11061417

**Published:** 2023-05-27

**Authors:** Hong Duc Thi Nguyen, Tan Minh Le, Eunmi Lee, Donghyeon Lee, Yeseul Choi, Junghwan Cho, Nora Jee-Young Park, Gun Oh Chong, Incheol Seo, Hyung Soo Han

**Affiliations:** 1 Department of Biomedical Science, Graduate School, Kyungpook National University, Daegu 41944, Republic of Korea; 2 BK21 Four Program, School of Medicine, Kyungpook National University, Daegu 41944, Republic of Korea; 3 Clinical Omics Institute, Kyungpook National University, Daegu 41405, Republic of Korea; 4 Department of Pathology, School of Medicine, Kyungpook National University, Daegu 41944, Republic of Korea; 5 Department of Pathology, Kyungpook National University Chilgok Hospital, Daegu 41404, Republic of Korea; 6 Department of Obstetrics and Gynecology, School of Medicine, Kyungpook National University, Daegu 41944, Republic of Korea; 7 Department of Obstetrics and Gynecology, Kyungpook National University Chilgok Hospital, Daegu 41404, Republic of Korea; 8 Department of Immunology, School of Medicine, Kyungpook National University, Daegu 41944, Republic of Korea; 9 Department of Physiology, School of Medicine, Kyungpook National University, Daegu 41944, Republic of Korea

**Keywords:** cervicovaginal microbiome, human papillomavirus, cervical cancer, cervical intraepithelial lesion, bacteriotherapy

## Abstract

Uterine cervical cancer (CC) is a complex, multistep disease primarily linked to persistent infection with high-risk human papillomavirus (HR-HPV). However, it is widely acknowledged that HR-HPV infection alone cannot account for the formation and progression of CC. Emerging evidence suggests that the cervicovaginal microbiome (CVM) also plays a significant role in HPV-related CC. Certain bacteria, such as *Fusobacterium* spp., *Porphyromonas*, *Prevotella*, and *Campylobacter*, are currently being considered as potential microbiomarkers for HPV-positive CC. However, the composition of the CVM in CC is inconsistent; thus, further studies are needed. This review comprehensively discusses the complex interplay between HPV and the CVM in cervical carcinogenesis. It is postulated that the dynamic interaction between HPV and the CVM creates an imbalanced cervicovaginal microenvironment that triggers dysbiosis, enhances HPV persistence, and promotes cervical carcinogenesis. Moreover, this review aims to provide updated evidence on the potential role of bacteriotherapy, particularly probiotics, in the treatment of CC.

## 1. Introduction

Uterine cervical cancer (CC) remains a major public health problem, with a significant number of new cases and deaths annually [1]. Persistent human papillomavirus (HPV) infection, particularly high-risk genotypes (HR-HPV), is the most common cause of CC [2]. It is undeniable that HR-HPV infection is essential for CC formation and progression, but not sufficient alone. Moreover, drivers of the transition state between HPV acquisition, spacing, and persistence are poorly understood [3].

The cervicovaginal microbiome (CVM) has been considered essential to the female vaginal flora [4]. In most healthy women, the CVM is dominated by *Lactobacillus* spp., which benefits the host through symbiotic relationships [5,6]. *Lactobacillus* spp. depletion can lead to CVM dysbiosis, which may enhance tumor development through several mechanisms, such as promoting chronic inflammation, dysregulating the immune system, and producing genotoxins [7].

Recently, mounting evidence has suggested an interaction between HPV, the CVM, and CC progression [8]. On the one hand, HPV infection can induce changes in the cervicovaginal microenvironment [9]. Consequently, this can lead to CVM dysbiosis and cancer. On the other hand, abnormalities in the cervicovaginal flora may change vaginal pH, release bacteriocin, and disrupt the mucosal layer. As a result, dysbiosis may contribute to HPV-related CC by interfering with HPV infection, binding, internalization, integration, gene expression, and telomerase activation [10]. Several studies have reported that women with HPV infection and CVM dysbiosis show high-grade squamous intraepithelial lesions (HSILs) or cancer [11,12], which may require closer follow-up and advanced treatment.

Bacteriotherapy, especially probiotics, has attracted enormous interest in CC prevention and treatment due to their antitumor activities [13,14,15,16]. Probiotics may become the best choice for controlling the complex interaction between the CVM, HPV, and cervical carcinogenesis.

To understand the complex role of microorganisms in cancer, this review aimed to comprehensively discuss the relationship between the CVM and HPV infection in CC carcinogenesis and update current research on bacteriotherapy in CC.

## 2. Human Papillomavirus

### 2.1. Structure and Genome

HPV is the most common cause of sexually transmitted infections in women. This virus belongs to a group of nonenveloped and double-stranded DNA viruses [17]. The HPV genome is circular DNA containing eight open reading frames and divided into three encoded regions: an early region encoding a nonstructural protein (E1, E2, and E4–E7) for replication, a late region encoding viral capsid proteins (L1 and L2) for viral assembly, and a long control region (LCR) or upstream regulatory region [18].

There are >200 types of HPV, and ~40 genotypes infect the mucosal epithelium in the anogenital tract. Based on the association between these types and carcinogenicity, they are categorized into low-risk HPVs (HPV6, 11, 40, 42–44, and 54) and HR-HPVs (HPV16, 18, 31, 33, 35, 39, 45, 51, 52, 56, 58, and 59) [19]. For example, HPV6 and HPV11, low-risk HPVs, are related to benign warts. In contrast, HPV16 and HPV18, HR-HPVs, can represent intraepithelial neoplasia with the potential for malignant progression [20].

### 2.2. Life Cycle of HPV

The most common HPV infections are caused by sexual intercourse when the vaginal and cervical epithelia are exposed to HPV through a microwound [21]. After entering basal keratinocytes, the viral genome moves to the nucleus and is maintained as episome DNA with a low copy number (50–100 copies per cell) [22].

In the early region, E2 is considered a regulator factor, whereas E6 and E7 are critical in inhibiting host tumor suppressor genes and oncogenic transformation [23]. Notably, the formation of an E1-E2 complex is required for the stable binding of the E1 helicase to the LCR ori site and controls the transcriptional levels of E6 and E7 viral oncogenes [24]. After integrating the HPV genome into the host DNA, the connection between E1 and E2 breaks, and E2 expression is lost. As a result, E6 and E7 expression is upregulated, which leads to the inactivation of tumor suppressor proteins p53 and pRb, respectively. This condition will promote malignant transformation in the cervix [25,26,27,28,29].

Until keratinocyte differentiation, the productive stage of the viral life cycle occurs by activating the late promoter (L1 and L2) and late viral gene expression (E4 and E5). Moreover, E1 and E2 expression increases HPV DNA amplification between 100 and 1000 episomal copies per cell [30,31]. Viral particles were then released from the uppermost layers of the stratified epithelium. There are ~3 weeks from infection to the release of the virus in which the virus can evade the immune system, and the appearance of lesions can occur after weeks to months [32].

### 2.3. HPV and Host Immune Responses

The host immune system, including the innate and adaptive immune systems, plays a vital role in clearing or controlling the infection and eliminating HPV-induced lesions.

#### 2.3.1. HPV and Innate Immune Response

The essential step of the innate immune response is to detect pathogen-associated molecular patterns (PAMPs) by receptors located on the surface of sensor cells. During the early stages of HPV infection, pattern recognition receptors (PRRs) can recognize HPV and activate a cascade of antiviral signaling pathways to defend against its invasion. These pathogen sensors can detect DNA (known as DNA sensors: absent in melanoma 2 [33], interferon (IFN)-γ inducible protein 16 [34,35], Toll-like receptor 9 [36], and cyclic GMP-AMP synthase [37,38]) or RNA (known as RNA sensors: Toll-like receptor 3 [36], retinoic acid-inducible gene I [39], and melanoma differentiation-associated gene 5 [40]) in the cytoplasm or nucleus. One of the most important downstream reactions is the induction of IFN signaling, including types I and III IFNs, which stimulates the Janus kinase (JAK)/signal transducers and activators of transcription (STAT) signaling cascade, enhances IFN-stimulated gene (ISG) expression, and results in HPV clearance [41,42,43,44].

Several types of immune sentinels (sensor cells) exist in the innate system, such as dendritic cells, Langerhans cells, natural killer (NK) cells, and keratinocytes. Among them, keratinocytes target cells of HPV in early infection and play an essential role in response to the recognition between PRRs and PAMPs. Keratinocytes can detect various HPV-related patterns and secrete various cytokines and chemokines. These cytokines and chemokines promote immune responses and recruit more immune cells to the HPV-related microenvironment [17,45].

#### 2.3.2. HPV and the Adaptive Immune Response

The adaptive immune system includes cell-mediated immune responses and antibody-mediated humoral immunity. The cell-mediated system is vital for destroying virus-infected cells, and the antibody-mediated system is responsible for clearing free pathogen particles from body fluids [46]. During HPV invasion, CD4^+^ T helper 1 (Th1) cells can detect HPV E6, E7, and E2 and induce cytotoxicity by activating CD8^+^ T cells and releasing interleukin (IL)-2 and IFN-γ [47]. It is agreed that whereas innate responses play a role in early HPV clearance, adaptive responses are essential to determining and eliminating HPV-induced lesions, particularly effector T cells [46].

A meta-analysis by Litwin et al. [48] supported the important role of T-cell populations in the outcome of cervical HPV infections. According to this study, helper and killer T cells are found at lower levels in low- and high-grade cervical lesions than in normal tissue, suggesting that the virus evades immune detection in patients with persistent lesions. Moreover, Foxp3^+^ and CD25^+^ regulatory T-cell (Treg) infiltration was high in precancerous HPV-related lesions, and longitudinal data showed improved outcomes with lower Treg levels.

#### 2.3.3. HPV and Immune Suppression

**Figure 1:**
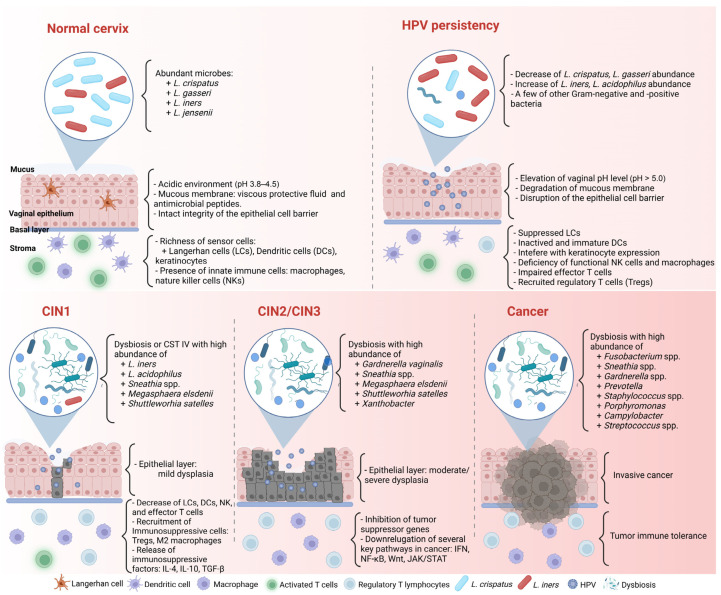
Complex association between HPV infection, the CVM, and cancer formation in the cervicovaginal microenvironment. CIN: cervical intraepithelial neoplasia. Created with BioRender.com (accessed on 30 March 2023).

Even if the immune reaction seems perfect and can clear most HPV infections, HPV has several immune evasion mechanisms and can facilitate cancer progression (Figure 1). First, most HPVs in the intraepithelial layer provide almost no viremia, release few proteins, induce less inflammation, and cause few host cell deaths [47,49]. Therefore, HPV can often prevent itself from being recognized by sensors.

Second, HPV can alter host gene, transcript factor, or protein expression to evade the immune response. For example, HPV blocks IFN signaling by inhibiting the activity of the IFN regulatory factors, interfering with the JAK/STAT signaling pathway, and downregulating the IFN-κ and ISG expressions. Moreover, HPV deregulates the activity of transcription factors, particularly nuclear factor-κB (NF-κB), to repress proinflammatory cytokine production. NF-κB is one of the members of the NF-κB family responsible for controlling cell proliferation and apoptosis through the NF-κB/IFN signaling pathway. HR-HPV can eradicate the inhibitory effect of the immune system and lead to persistent infection by decreasing NF-κB activation [50,51].

Third, the HPV-related microenvironment can inhibit the infiltration of helper and cytotoxic T cells while recruiting more immunosuppressive cells, such as M2 macrophages, myeloid-derived suppressor cells (MDSCs), and Tregs [48,52]. Tregs are related to downregulated responses to exogenous antigens. When Tregs are recruited to a tumor, they suppress the adaptive immune response and promote HPV-related lesion formation. M2 macrophages and MDSCs can enhance HPV-associated tumor progression by inhibiting CD4^+^ and CD8^+^ T cells, attracting Tregs, and producing immunosuppressive factors such as IL-10 and transforming growth factor-β (TGF-β) [53].

## 3. Cervicovaginal Microbiome

### 3.1. Cervicovaginal Microbiome in Healthy Women

*Lactobacillus* plays a critical role in cervicovaginal health in women [54]. They protect the vagina from bacterial invasion by maintaining an acidic environment and promoting the integrity of the epithelial cell barrier and intercellular junctional proteins [5,55,56].

Based on next-generation sequencing, many human microbiota studies have identified complex microbial communities in healthy women [54,57,58]. Ravel et al. [4] utilized 16S rRNA sequencing on 396 asymptomatic women of different ethnicities and investigated five community state types (CSTs). CSTs I to III and V present low diversity and are dominant with *Lactobacillus crispatus*, *Lactobacillus gasseri*, *Lactobacillus iners*, and *Lactobacillus jensenii*, respectively. In contrast, CST IV is characterized by reduced lactobacilli abundance and high diversity with a significant quantity of vaginosis bacteria, such as *Gardnerella vaginalis*, *Megasphaera*, *Sneathia*, and *Prevotella* spp.

The CVM is dynamic; one CST can transform into another in the same healthy woman. For example, an *L. crispatus*-dominated community often changes to dominant *L. iners* or mixed lactobacilli, and the *L. iners*-dominated one likely transforms into a bacterial vaginosis (BV)-associated community (CST IV) [59].

The CVM composition is influenced by numerous factors, such as ethnicity, hormonal status, sexual activity, age, menstrual cycle, or menopause [5,60,61]. Women of different ethnic groups are associated with different CVMs. For example, *L. iners* and *L. crispatus* were the most abundant cervicovaginal microbial communities in healthy Asian women [62], whereas *L. iners* and CST IV were dominant microbial communities in Black and Hispanic women [4].

In reproductive-aged women, the cyclic secretion of hormones determines significance, especially during the menstrual phase. In Cheng et al.’s study on Chinese women’s vaginal microbiota at childbearing age [63], *L. iners* and *Lactobacillus helveticus* are the most abundant species in both follicular and luteal phases [64]. Furthermore, low estrogen and progesterone levels and existing menstrual blood in the female vagina are related to the decrease in some microorganisms and the enrichment of others. Accordingly, during menses, reproductive-aged women may have a low amount of *Lactobacillus* spp. and a high abundance of other species, such as *G. vaginalis*, *Streptococcus* spp., and *Anaerococcus* spp. [65]. The reason is that the high iron level in menstrual blood may enhance the growth of bacteria such as *G. vaginalis* [66].

To understand the association between the CVM and several female conditions (pregnancy history, marriage, number of vaginal deliveries, age at first instance of vaginal sex, and breastfeeding), Jie et al. [57] utilized metagenomic shotgun sequencing on cervical samples from 516 women and recorded their life histories through questionnaires. They revealed that the cervicovaginal microbiota might reflect female physical status. For instance, *L. crispatus* was significantly higher in women with fewer pregnancies and overrepresented in menstruation. However, its abundance was depleted during breastfeeding or postmenopause.

### 3.2. Cervicovaginal Microbiome, Host Response, and Cervical Cancer

The CVM with predominant *Lactobacillus* protects the female genital tract against bacterial pathogens [5]. When lactobacilli are depleted, invading microorganisms can easily penetrate epithelial cell layers. Hence, a CVM with nondominant *Lactobacillus* and increased pathogenic microbial diversity, such as *G. vaginalis*, *Atopobium*, *Prevotella*, *Sneathia*, and *Megasphaera*, is commonly seen in BV or dysbiosis [67,68,69,70,71]. BV can damage the mucus and cytoskeleton structures, increase cell death, change antimicrobial peptides [62], and promote proinflammatory cytokine production [72,73,74,75].

Anahtar et al. [76] investigated a group of asymptomatic women in South Africa and found that most women had low *Lactobacillus* abundance and a high diversity of bacterial communities. *Sneathia sanguinigens*, *Sneathia amnii*, *Mobiluncus mulieris*, and *Prevotella amnii* in high-diversity communities are related to the presence of genital proinflammatory cytokines, such as IL-1α, IL-1β, and IL-8. Furthermore, the bacterial lipopolysaccharide in the CVM can be sensed by antigen-presenting cells on epithelial cells, triggering NF-κB pathways and Toll-like receptors and the recruitment of lymphocytes through chemokine secretion. Laniewski et al. [77] found similar results in 3D epithelial cervical cell models co-cultured with *Lactobacillus crispatus* and BV-associated bacteria *Gardnerella vaginalis*, *Atopobium vaginae*, *Prevotella bivia*, and *Sneathia amnii*. Specifically, *Lactobacillus crispatus* enhanced protection of the cervical microenvironment through antimicrobial metabolites, including decreasing glucose and the production of phenyllactate and N-acetylated amino acids in 3D cervical models. In contrast, *Atopobium vaginae* and *Sneathia amnii* induced the greatest proinflammatory cytokines (IL-6, IL-8, TNFα, etc.), iNOS, and oxidative stress, while *Gardnerella vaginalis*, *Prevotella bivia*, and *Sneathia amnii* altered the epithelial barrier by decreasing protein and metabolite levels, such as mucins, sialic acid, and polyamines [77].

These alterations in the inflammatory cytokine profile present in a CVM environment may result in chronic inflammation, a risk factor for cervical carcinogenesis [73].

In addition, Fan et al. [78] reported the relationship between CVM dysbiosis and mucosal epithelial cell fucosylation, which is a protective component of vaginal epithelial cells [78]. Knocking out the core fucosyltransferase gene can promote CC cell proliferation and invasion. *L. iners* metabolites can increase core fucosylation levels by activating the Wnt pathway and inhibiting CC proliferation and migration. Hence, CVM imbalance and a low abundance of *L. iners* may result in abnormal fucosylation, which promotes the development of CC.

## 4. HPV Infection, Cervicovaginal Microbiome, and Cervical Carcinogenesis

The interaction between the host and microorganisms in cancer, particularly CC, is complex and poorly understood. Nevertheless, recent studies have provided critical evidence and insights into this vital field [79].

### 4.1. HPV Infection and Cervicovaginal Microbiome

HPV infection status is closely related to cervicovaginal dysbiosis (Figure 1) [9,70]. Di Paola et al. [80] collected cervicovaginal samples from 55 HPV-positive women in Italy to identify the CST related to persistent HPV [80]. To fulfill their purpose, they followed up with the candidates and checked the clearance or persistence of HPV after 12 months. More than 40% of persistent HPV-positive women presented in the CST IV subgroup with *Gardnerella*, *Prevotella*, *Atopobium,* and *Megasphaera*. Significantly, *Gardnerella* may contribute to the ongoing HPV status by secreting the sialidase enzyme involved in biofilm formation [80,81]. The association between biofilm formation in the CVM and HPV infection was confirmed by Donmez et al. [82]. In line with these results, Qingqing et al. [83] identified a high abundance of anaerobes, including *Prevotella*, *Sphingomonas*, and *Anaerococcus*, related to persistent HPV and a higher presence of Tregs, MDSCs, IL-6, and TNF-α in cervical secretions.

In addition, many studies have reported the association between the CVM and HR-HPV infection [3,84,85,86]. Most revealed a decrease in *Lactobacillus* spp. and an increase in CVM diversity in HR-HPV-positive women than in HR-HPV-negative women. For instance, Brotman et al. [87] identified that most HR-HPV-positive groups had a four-fold lower abundance of *Lactobacillus* spp., including *L. crispatus*, *L. jensenii*, and *L. gasseri,* compared to HPV-negative groups. In particular, Lee et al. [88] compared the differences in the CVM between HR-HPV-negative and HR-HPV-positive women in a Korean twin cohort. They proved that the HPV-positive group had higher species diversity and a lower abundance of *Lactobacillus* than their uninfected twins. Furthermore, they suggested that *Sneathnia* spp. could be identified as a microbiological marker of an HR-HPV infection. Also, Lebeau et al. [9] followed up on >6000 patients regarding their HPV infection and BV status for >8 years. By applying multiple analyses from patients, cell line cultures, and transgenic mouse samples, they discovered that the downregulation of NF-κB and Wnt/β-catenin signaling pathways due to persistent HPV infection can lead to the inhibition of most antimicrobial peptides, such as S100A7, SLPI, Elafin, HD6, HβD2, and HD5, and impair TNF-α/LPS. Because several antimicrobial peptides are the amino acid source for *Lactobacillus* survival, the *Lactobacillus* concentration was significantly decreased. In other words, the escape of HPV from the immune response results in an imbalance of microbiota in the female vaginal flora.

### 4.2. Cervical Carcinogenesis in the Relationship between HPV Infection and the Cervicovaginal Microbiome

HPV-persistent infection can interfere with the cervical microenvironment, cell proliferation, angiogenesis, and tissue differentiation. Any changes in regulatory factors in these processes may cause cervical neoplasia. However, HPV infection alone does not explain the development of CC. Indeed, some aspects, such as the milieu of mucosal secretion, epithelial surface integrity, immune regulation, and local microbiota, may influence HPV carcinogenesis [47]. Notably, increasing CVM diversity has been investigated to be associated with CIN progression and may be involved in regulating persistent HPV infection (Table 1) [89,90,91]. Guo et al. [11] identified differential microbial communities among 149 women with different HPV and SIL statuses. A non-*Lactobacillus* CVM was predominant in SIL women compared to HPV-negative and HPV-positive non-SILs. *S. oralis* and unclassified OTU265 differed between HPV-positive and HPV-positive LSILs. Also, this study revealed several unclassified OTUs, such as OTU880, OTU893, and OTU883, predominant in the HPV-positive HSIL group. In a longitudinal study, Usyk et al. [92] evaluated the CVM of 273 HR-HPV-infected women and tumor progression after two visits. They clustered the CVM into four CSTs: two CSTs related to *L. iners* and *L. crispatus*, one cluster containing high levels of *G. vaginalis*, and other CSTs without a significant group. *L. iners* were associated with HPV clearance, whereas *G. vaginalis* correlated with CIN2 progression after the first visit. *Prevotella amnii* and *Anaerococcus prevotii*, two common causative agents of BV, were linked to disease progression on the second visit. *L. iners* may have two opposite functions in the cervicovaginal microenvironment: one can promote health, and the other is associated with dysbiosis and CIN susceptibility [93]. For example, Oh et al. [94] identified a high abundance of *L. crispatus* in low-risk CIN, whereas *L. iners* was dominant in medium-risk CIN.

Concerning ICC, evidence about the relationship between the microbial community and ICC was also observed in several studies (Table 1). Audirac-Chalifour et al. [85] discussed the dynamics of the CVM depending on HPV status and cervical neoplasia lesions. The CVM with dominant *L. crispatus* changed into *L. iners* after HPV infection in the normal cervical microenvironment. When epithelial lesions became SILs, *Sneathia* and other *Fusobacterium* spp. were the most abundant. *Fusobacterium necrophorum* was highly represented in HPV-positive ICC cases and associated with increasing anti-inflammatory cytokines, such as IL-4 and TGF-β1. The authors concluded that *Fusobacterium* spp. might play a role in shifting Th1 immunity to Th2 or by having a direct effect on the E-cadherin/β-catenin signaling pathway in cervical HPV-transformed cells. However, this finding differed from Wu et al. [95], who found that *Fusobacterium* was not an excellent prognostic marker for CC, although the abundance of this bacteria was the highest among the groups. The potential marker genera for CC in this study were *Porphyromonas*, *Prevotella*, and *Campylobacter*. Together, the CVM composition in CC differed from case-to-case; therefore, a large and well-designed study on the microbiome in CC with different HPV statuses is necessary for clinical applications.

Recently, multiomics has become an increasingly important area in cancer research. Several multiomics studies were carried out and contributed various insights into the underlying mechanism of the complex interaction between CC and its microorganisms. IIhan et al. [96] evaluated the CVM metabolomic and metagenomic profiling of 78 women in Arizona. Amino acid and nucleotide metabolism disruptions were identified in non-*Lactobacillus*-dominant communities and in high-grade dysplasia. 3-hydroxybutyrate, eicosenoate, and oleate/vaccinate are the metabolic features of CC [96]. Another multiomics study also highlighted that pipecolate and deoxycarnitine were significantly related to vaginal dysbiosis with HPV infection. Particularly, 3-hydroxybutyrate was strongly associated with a high abundance of *Streptococcus*, *Prevotella*, *Megasphaera*, *Atopobium,* and *Sneathia* [97].

## 5. Bacteriotherapy in Cervical Cancer Treatment

### 5.1. Probiotic Bacteriotherapy in Cervical Cancer Treatment

In the early stages of CC, there are multiple choices for treatment, such as surgery (e.g., fertility preservation surgery), radiation, and neoadjuvant chemotherapy. In the locally advanced stage, chemoradiation is the best approach. However, 30–40% of the patients do not respond entirely, and some patients face side effects from this therapy [98]. Therefore, it is necessary to find a more effective and nontoxic or less toxic treatment [99].

Recently, bacteriotherapy has emerged as a promising platform for cancer treatment. One of the most common representatives of bacteriotherapy is probiotics. Probiotics contain a number of live microorganisms that provide several benefits to their host. There are many sources of probiotics in the human diet, such as fermented milk products (yogurt, cheese, and beer) and mainly vegetables (cabbage and cucumber) [100,101]. The most common genera of probiotics in the human diet are *Lactobacillus*, *Bifidobacterium*, *Lactococcus*, *Streptococcus*, and *Enterococcus*. Also, some strains belonging to *Bacillus* and *Saccharomyces* are utilized nowadays [102].

The past decade has seen the rapid development of probiotics for CC [10,14,103]. Researchers believed that probiotics could promote cancer cell apoptosis and inhibit tumor cell proliferation and metastasis [99]. As a result, probiotics can be used as an additional agent for enhancing or modulating other diagnostic and therapeutic methods. Moreover, probiotics can enhance HPV clearance through three hypotheses. First, an increased number of probiotic strains in the vagina may be able to prevent and reduce HPV infections by competing for space or nutrition and releasing several inhibitory factors, such as lactic acid, bacteriocins, biosurfactants, and aggregation molecules. An acidic environment can defend against pathogen invasion and growth [10]. Second, promoting an immune response is the principal mechanism against viral infections [104]. Last, the direct elimination of viruses occurs through the secretion of specific metabolites [105].

Various studies have assessed the efficacy of probiotics on CC cell lines. For instance, on the effect of *L. crispatus*, *L. jensenii*, and *L. gasseri* on CaSki cells, Wang et al. [106] reported that these subgenera of *Lactobacillus* could inhibit CC cell proliferation through the regulation of HPV oncogenes and cell cycle-related genes. Another study showed that *L. gasseri* could promote apoptosis in HeLa cells through its exopolysaccharides. Also, *L. gasseri* could affect the anti-inflammation of HeLa cells by reducing TNF-α and increasing the cytokine IL-10 [107]. Moreover, lactobacilli and their metabolites are essential in CC prevention and treatment. Pawar et al. [108] found that cell-free culture supernatants of 12 *Lactobacillus spp.* from different microenvironments resembled inhibitors of HPV 16 and 18 by restoring E-cadherin and suppressing MMP9. Another strain, *Bifidobacterium adolescentis* SPM1005-A, has potential antiactivity by inhibiting E6 and E7 oncogene expression in SiHa cells [103].

There are also reports on the special role of probiotics in HPV infection and CC in human trials (Table 2). In 2013, a prospective controlled pilot study in women with HPV infection and LSILs who took an oral probiotic product (*Lactobacillus casei*) for six months had higher HPV clearance than the control group; however, the difference was not significant [14]. In a well-designed study by Out et al. [109], 121 HPV-positive women were given oral-specific strains of *Lactobacillus rhamnosus* and *Lactobacillus reuteri* daily. Although the trial effects did not influence HPV clearance, they significantly decreased the rates of mildly abnormal and unsatisfactory cervical smears. Likewise, Dellino et al. [105] found that HPV-positive women who took the long-term oral probiotic *L. crispatus* M247 had reduced HPV-related cytological anomalies compared to the control group. However, HPV clearance was not significantly different between the probiotic group and the group without probiotics. In contrast, Pierro et al. [15] reported an increase in HPV clearance after 90 days of taking *L. crispatus* M247; furthermore, in some cases, the CVM shifted the CST status to CST I.

### 5.2. Novel Approaches Using Bacteria in Cervical Cancer Treatment

#### 5.2.1. Vaginal Suppositories

In addition to oral probiotics, vaginal suppositories containing *Lactobacillus* have been studied and developed to prevent vaginal dysbiosis, such as BV or vulvovaginal candidiasis (Table 3) [110,111]. In a clinical trial, Tomusiak et al. [111] revealed that vaginal medicinal therapy (containing *Lactobacillus fermentum* 57A, *Lactobacillus plantarum* 57B, and *L. gasseri* 57C) is safe and can promote an increased abundance of *Lactobacillus* and decreased pH and Nugent score in women with symptomatic BV after four visits. Until now, there have been limited studies using probiotic vaginal administration in CC treatment, but some vaginal suppositories have reported high efficacy in HPV-positive women. A vaginal *L. rhamnosus* BMX therapy was implemented in 117 women with HPV and BV or vaginitis concomitantly after using an antibiotic (metronidazole) or antifungal (fluconazole) long-term. The study revealed that vaginal *L. rhamnosus* BMX could change abnormal cervical lesions and increase HPV clearance after long-term therapy [112].

Also, lacidophilin, a bacteriocin produced by lactic acid bacteria, has been considered a promising therapy. Lacidophilin can be an antibacterial factor by generating lactic acid and destroying the bacterial membrane [113]. The combination of lacidophilin and antitumor IFN-α2b was investigated for treating HPV-infected patients, with significant results in recovering vaginal microecology and inhibiting inflammatory factors [114].

The combination of probiotics and chemotherapy is currently being reported as a new approach to cervical cancer (CC) treatment. Previous studies have shown that using probiotic supplements can reduce the side effects or toxicity of chemotherapy [115]. For instance, cisplatin is a common therapy for CC; however, its toxicity affects normal cells and tissues. Negi et al. [116] combined cisplatin with probiotic-loaded pessaries (Lactobacillus rhamnosus) in a vaginal mouse model. They found a better outcome with fewer side effects of cisplatin and a reduced tumor volume in the treated group.

#### 5.2.2. Probiotic Injection

A recent study reported the combination of a heat-killed preparation of *L. casei* and α-GalCer (an anticancer and NK T-cell stimulator) subcutaneous injections in a mouse model of CC. They found that splenocyte proliferation, lactate dehydrogenase, nitric oxide, and IFN-γ levels increased more in the combination therapy group than in the control group. Compared to Gardasil injection in the mouse model, the heat-killed preparation of *L. casei* and α-GalCer had a similar effect. These results could promise a new therapy for CC management [117].

#### 5.2.3. Probiotics and Modulating the Gastrointestinal Problem of Cervical Cancer

Diarrhea is the most common side effect of radiotherapy in treating cervical cancer (CC) patients [118]. Previous studies have suggested that the supplementation of probiotics may prevent this gastrointestinal problem. For instance, Linn et al. [119] investigated the efficacy of *Lactobacillus acidophilus* LA-5 and *Bifidobacterium animalis* subsp. *lactis* BB-12 in 57 CC patients with diarrhea after radiotherapy. The study found that diarrhea symptoms in the mild-to-moderate group and the severe group were significantly reduced after three weeks of using probiotics.

#### 5.2.4. Vaginal Microbiota Transplantation (VMT)

VMT may improve CVM imbalance when transplanting vaginal fluid from a healthy person with high *Lactobacillus* abundance [120]. Lev-Sagie et al. [121] reported applying VMT to intractable and recurrent BV treatment. Five patients used VMT, and four had remissions with symptom improvement and no side effects after 5–21 months of transplantation. Although the number of patients was too small, this study could improve CVM dysbiosis and CC management in the future.

Overall, these findings suggest a significant role for probiotics in fighting CC. Nevertheless, further studies are required to understand the function of bacteriotherapy and its underlying mechanisms, particularly during CC treatment.

**Table 3:** Novel approaches using bacteria for HPV in cervical cancer treatment.

Studies	Participate	Probiotics	Administration	Results
Sun et al. (2022) [114]	200 HPV-infected women (90 control group, 110 research group)	Lacidophilin + rhIFN-α2b	Vaginal capsules	HPV-positive decreased, and the vaginal microecology recovered higher in the research group than using rhIFN-α2b alone.
Palma et al. (2018) [112]	117 women (BV or vaginitis with concomitant HPV infections)	*L. rhamnosus* BMX 54 (after treatment with metronidazole for 7 days or fluconazole for 2 consecutive days)	Vaginal tablets, short-term (3 months) and long-term (6 months), follow-up for 9–30 months	HPV-related cytological abnormalities decreased in the long-term vaginal probiotic group. HPV clearance increased after using vaginal probiotics.
Negi D. et al. (2020) [116]	Mouse model of CC	*L. rhamnosus + cisplastin* -loaded pessaries	Vaginal pessaries	Reduced the tumor volume in mice with CC and has a low side effect of cisplatin.
Haghighi et al. (2022) [117]	Mouse model of CC with TC1 cells	*L. casei* combined to α-GalCer	Heat-killed extracts of *L. casei* and α-GalCer (subcutaneous injections)	Increasing splenocyte proliferation and cell death and decreasing IL-4 and TGF-β. The combination can be efficacious in the CC treatment model.
Linn et al. (2019) [119]	57 CC patients with diarrhea after radiotherapy	*Lactobacillus acidophilus* LA-5 and *Bifidobacterium animalis* subsp. *lactis* BB-12	Oral capsule (Biogurt^®^)	The diarrhea symptoms were significantly reduced.
Le-Sagie et al. (2019) [121]	5 patients with intractable and recurrent BV	Vaginal microbiota transplantation	Vaginal health fluid from donors	Four patients improved their symptoms, the Amsel score normalized, and microscopic vaginal fluid appeared.

## 6. Conclusions

This review suggests a strong association between HPV infection and CVM status in cervical diseases. Accordingly, the dynamic interaction can cause an imbalanced cervicovaginal microenvironment, such as depletion of *Lactobacillus* and changes in the immune system and metabolism, which induce dysbiosis, enhance HPV persistence, and promote cervical carcinogenesis. However, this research is only beginning to understand the role of microorganisms in carcinogenesis. Further studies combining new molecular tools, or multiomics, should be considered to identify underlying mechanisms and provide insights into the interaction of microorganisms with immune and metabolic responses. Notwithstanding the relatively limited evidence, probiotics offer new opportunities for future therapy that could change the response to cancer treatment. Hence, continuing well-designed studies about the antitumor function of probiotics in managing CC patients is necessary.

## Figures and Tables

**Table 1:** Association between the cervicovaginal microbiome and HPV in cervical neoplastic lesions.

Author	Participants	Sample Types	Microbial Analysis	* Healthy	* CIN1 or LSIL- HPV(+)	* CIN2/3 or HSIL- HPV(+)	* ICC
Audirac-Chalifour et al. (2016) [85]	32 Mexican women	Cervical epithelial scraping swabs, fresh biopsy	V3-V4 regions of 16S rRNA	*L. crispatus*, *L. iners*	*Sneathia* spp., *Megasphaera elsdenii*, *Shuttleworhia satelles*	*Sneathia* spp., *M. elsdenii*, *S. satelles*	*Fusobacterium* spp.
Kwasniewski et al. (2018) [89]	250 Polish women	Cervical swabs	V4 region of 16S rRNA	*L. crispatus*, *L. iners, L. taiwanensis*	*L. acidophilus*, *L. iners*	*G. vaginalis*, *L. acidophilus*	
Laniewski et al. (2018) [73]	100 Hispanic women (USA)	Cervical (swabs, lavage)	V4 region of 16S rRNA	*Lactobacillus* spp.	*L. iners*	*L. iners*	*Sneathia* spp.
Kang et al. (2021) [12]	23 Korean women	Cervicovaginal swabs	V3 region of 16S rRNA	*Lactobacillus* spp.		*Gardnerella*, *Prevotella*	*Streptococcus* spp.
Wu et al. (2021) [95]	94 Chinese women	Cervical mucus discharge	V4 region of 16S rRNA	*Lactobacillus* spp.	*Lactobacillus*, *Xanthobacter*, *Thermus*, *FlavisolibacterSphingopyxis*, *Sediminibacterium*, *Geobacillus*	*Sneathia, Gardnerella*, *Megasphera*	*Porphyromonas*, *Prevotella*, and *Campylobacter*
Teka B. et al. (2023) [90]	120 Ethiopian women	Cervical (swabs, brush)	V4 region of 16S rRNA	*Lactobacillus* spp.	*L. iners*	*L.iners*	*Porphyromonas somerae, Prevotella timonensis, Porphyromonas asaccharolytica*

LSIL: low squamous intraepithelial lesions; ICC: invasive CC; * key bacterial communities.

**Table 2:** Treatment effects of *Lactobacillus* spp. on HPV and cervical cancer in clinical trials.

Studies	NCT Number	Participants	Strain	Treatment	Results in CC	HPV Clearance
Veronique et al. (2013) [14]	NCT01097356	54 women with HPV-positive LSILs	*L. casei* strain Sheroni (Yakult)	Oral daily for 6 months	Probiotics significantly contributed to the resolution of cytological abnormalities	HPV clearance was higher in probiotic takers than in the control group, but not statistically significant
Out et al. (2019) [109]	NCT01599416	121 women 62 HR-HPV(+), 59 control	*L. rhamnosus* GR-1 *L. reuteri* RC-14	Oral daily until HPV-	Decreased, mildly abnormal, and unsatisfactory cervical smears in group probiotic takers	Noninfluence HR-HPV
Pierro et al. (2021) [15]	Not specified	35 HPV-positive women	*L. crispatus* M247	Oral, 90 days	Change in CST status to CST I after using probiotics	Increased HPV clearance in group probiotic takers
Dellino et al. (2022) [13]	Not specified	80 women with HPV infection and 80 control	*L. crispatus* M247	Oral, follow-up 12 months	Reducing HPV-related cytological anomalies in long-term oral probiotic users	The proportion of HPV-negative women was not significantly different

LSIL: low squamous intraepithelial lesions; HR-HPV: high-risk HPV.

## Data Availability

Not applicable.
